# From Llama to language: prompt-engineering allows general-purpose artificial intelligence to rate narratives like expert psychologists

**DOI:** 10.3389/frai.2025.1398885

**Published:** 2025-02-06

**Authors:** Barry Dauphin, Caleb Siefert

**Affiliations:** ^1^Department of Psychology, University of Detroit-Mercy, Detroit, MI, United States; ^2^Behavioral Sciences, Psychology, University of Michigan-Dearborn, Dearborn, MI, United States

**Keywords:** Social Cognition and Object Relations Scales, artificial intelligence, narrative research, psychopathology, personality, personality assessment

## Abstract

**Introduction:**

Artificial intelligence (AI) has tremendous potential for use in psychology. Among the many applications that may benefit from development of AI applications is narrative-personality assessment. Use of these tools and research methods is notably time-consuming and resource intensive. AI has potential to address these issues in ways that would greatly reduce clinician and researcher burden. Nonetheless, it is unclear if current AI models are sufficiently sophisticated to perform the complex downstream tasks, such as narrative assessment.

**Methodology:**

The purpose of this study is to explore if an expert-refined prompt generation process can enable AI-empowered chatbots to reliably and accurately rate narratives using the Social Cognition and Object Relations scales – Global Rating Method (SCORS-G). Experts generated prompt inputs by engaging in a detailed review of SCORS-G training materials. Prompts were then improved using an systematic process in which experts worked with *Llama-2-70b* to refine prompts. The utility of the prompts was then tested on two AI-empowered chatbots, *ChatGPT-4* (OpenAI, 2023) and *CLAUDE-2-100k*, that were not used in the prompt refinement process.

**Results:**

Results showed that the refined prompts allowed chatbots to reliably rate narratives at the global level, though accuracy varied across subscales. Averaging ratings from two chatbots notably improved reliability for the global score and all subscale scores. Experimentation indicated that expert-refined prompts outperformed basic prompts regarding interrater reliability and absolute agreement with gold standard ratings. Only the expert-refined prompts were able to generate acceptable single-rater interrater reliability estimates.

**Discussion:**

Findings suggest that AI could significantly reduce the time and resource burdens on clinicians and researchers using narrative rating systems like the SCORS-G. Limitations and implications for future research are discussed.

## 1 Introduction

Rapid advancements in artificial intelligence (AI) have several applications for psychology and healthcare. AI-powered chatbots, such as *ChatGPT-4* (OpenAI, 2023), and *CLAUDE-2-100k* (Anthropic, 2023), are seeing widespread adoption (Grudin and Jacques, 2019). Humans can prompt chatbots with natural language to successfully perform a wide range of downstream tasks chatbots were not specifically developed to complete (Copeland, 2023). Developing and improving prompts can enable AI chatbots to assist or even perform healthcare related tasks (Meskó, 2023). However, prompts are not always sufficient to enable AI-models or chatbots to complete tasks requiring a high degree of expertise, understanding of technical jargon, or awareness of decision-making rules (Sun and Zhou, 2023). For such tasks, sophisticated AI-training techniques, such as machine learning, deep learning, and fine-tuning, may be necessary, especially in healthcare or research settings (Maharjan et al., 2024; Shah, 2023). Nonetheless, the highly advanced pretraining process of widely available AI-empowered chatbots is impressive, especially regarding completing language-intensive tasks. Thus, users should not immediately assume that sophisticated training techniques are required for all complex tasks.

The present study examines whether expert-generated and -refined prompts enable chatbots to rate narratives using a highly sophisticated narrative-assessment system routinely used in clinical psychology and personality research, the Social Cognition and Object Relations scales – Global Rating Method (SCORS-G; Stein and Slavin-Mulford, 2018, Westen, 1991). We examine if expert-refined prompts enable chatbots to reliably rate narratives (relative to gold standard ratings provided by human experts). We also explore if expert-refined prompts outperform more basic prompts.

### 1.1 The potential for artificial intelligence applications in psychology and narrative assessment

AI-empowered chatbots, with their capacity to make language inferences (Copeland, 2023), hold significant potential for applications in clinical psychology and psychological research (Adamopoulou and Moussiades, 2020; Bzdok and Meyer-Lindenberg, 2018; Elyoseph et al., 2023). The integration of AI could greatly enhance narrative assessments (Koutsouleris et al., 2022), a form of assessment that involves analyzing features of narratives to identify behavioral, emotional, and cognitive patterns. Narrative assessments require detailed, subjective analyses by trained experts. Narrative assessment systems have been developed for assessing personality features, motivation, and attachment (George and West, 2001; McAdams, 2012; Smith et al., 1992; Stein and Slavin-Mulford, 2018). Developing and refining these tools requires extensive, resource-intensive research. Human raters must undergo significant training to achieve sufficiently reliable results. Teams of raters are needed to code hundreds of narratives, further extending the timeline. Narrative assessment is notably burdensome, and studies in this area lag far behind self-report research.

AI has the potential to transform narrative assessment practices, as AI may be able to reliably rate narratives faster than human raters. This could significantly reduce the time needed for research, allowing studies to be conducted in weeks rather than months or years. AI may also be able to efficiently code large datasets, addressing challenges faced by human raters, such as rater drift, fatigue, and attrition. By rapidly processing extensive narrative data, AI could ease the workload for psychologists and enhance the detection of complex patterns, potentially uncovering insights that human observers might miss (Khurana et al., 2023).

While enthusiasm for AI-raters is understandable, skepticism remains (Minerva and Giubilini, 2023). It is unclear whether AI-empowered chatbots can use pretraining to infer reliable and valid narrative ratings. Most psychological tasks, including narrative assessment, require a deep understanding of psychological theory, semantics, grammar, and symbolic language. It is unclear whether chatbot pretraining gives them the sophistication necessary to assess narratives like expert psychologists. Chatbot performance, however, is not solely determined by pretraining. It is impacted by the quality of prompt inputs used to direct them. For a task as complex as narrative assessment, it is unlikely that simple prompting strategies will prove effective. Instead, prompts likely need to be optimized by experts.

### 1.2 Prompt optimization and assessment for complex downstream tasks

Prompting refers to the process of crafting phrases or templates that provide pretrained AI models or chatbots with input text that seeks to allow them to complete a downstream task that they were not originally trained to perform (Emerson, 2023). Prompt optimization seeks to enhance a chatbot’s capabilities to perform a task without directly manipulating underlying algorithms or parameters. Prompt optimization with AI-empowered chatbots offers numerous benefits. It requires no coding, avoids complex machine learning procedures, and allows for easy deployment. Prompt optimization has been successfully used in psychology to enhance chatbot performance on categorization tasks, such as depression and emotion detection (Priyadarshana et al., 2024).

Prompt optimization stands in contrast to fine tuning procedures that employ machine learning. Fine-tuning in machine learning involves providing a pre-trained AI model with data in order to improve its performance on a specific task that requires a high level of expertise or understanding of jargon. This process alters the model’s underlying parameters and seeks to improve the models ability to understand nuanced patterns relevant to the task. Like prompt engineering, this process may or may not be successful. Researchers must test the model against performance of human experts. Fine-tuning a machine learning model can be resource-intensive, requiring significant computing power. It risks overfitting, meaning the model might become too specialized and lose its ability to generalize to other datasets or tasks. Fine-tuning requires technical expertise, making it less accessible for psychologists. Prompt engineering and optimization is more flexible and user-friendly, allowing for quick adjustments without altering the model itself (Shah, 2023). Fine-tuning does not always prove superior performance to prompt-engineering, even for complex tasks. Maharjan et al. (2024) found that state-of-the-art generalist AI models, like GPT-4 and Gemini, surpassed fine-tuned models on medical question-answering tasks across several benchmarks with optimized prompts. Similar results have been observed in areas such as symbolic reasoning and clinical note classification (Nori et al., 2023; Wei et al., 2022; Zhang et al., 2024). General purpose AI models often possess superior language inference abilities owing to more extensive pre-training and possession of substantially more parameters relative to publicly available, open-source LLMs that may be downloaded for fine-tuning (Maharjan et al., 2024). It is possible that prompt-optimization could empower chatbots to effectively rate narratives using well-validated systems. This entails assessment of prompt effectiveness.

Prompt effectiveness is typically assessed in multiple ways. First, ratings generated by AI chatbots using prompts are compared to gold-standard, ground truth ratings to assess reliability. Effectiveness is also assessed by comparing chatbot performance across different prompting levels. For basic tasks, vanilla prompts or zero-shot prompting—providing a simple instruction like “Tell me a joke”—can be often sufficient. For more complex tasks, prompts may include one (i.e., one-shot prompting) or more examples (i.e., multiple-shot prompting) to demonstrate how to categorize a narrative or stimulus. When tasks involve multiple steps, chain-of-thought prompts and/or expert-refined prompts may outperform one-shot or multiple-shot approaches. Chain-of-thought prompting breaks down complex tasks into manageable steps, while expert-crafted prompts provide detailed instructions developed through an iterative process. Expert crafted and refined prompts aim to mitigate potential biases or errors stemming from a model’s pre-training (Gao et al., 2019; Liu et al., 2021; Ribeiro et al., 2020). These forms of prompt optimization have been useful in psychology for enhancing large language models’ ability to detect conditions like anxiety and depression (Englhardt et al., 2024) and assess quality of thought (Chen et al., 2023). For complex tasks, expert involvement in necessary to optimize prompts (Copeland, 2023; DAIR.AI, 2024; Lester et al., 2021). Narrative rating systems involve several rules for assigning ratings. Thus, expert involvement is necessary.

### 1.3 The Social Cognition and Object Relations – Global Rating Method

The SCORS-G is utilized by psychologists in psychological assessment, treatment planning, and progress monitoring. It is also used by researchers to study personality. The SCORS-G provides a reliable and valid tool for rating stories, relationship episodes, psychotherapy narratives, and autobiographical memories (see Stein and Slavin-Mulford, 2018).

We selected the SCORS-G for our study because it enables us to assess chatbot performance at various levels. The SCORS-G produces a global score, for overall object relations, and eight subscale scores assessing specific capacities (see Table 1 for names, abbreviations, and descriptions of each subscale). This allows us to evaluate overall reliability ratings for the global scale and explore the utility of prompts for assessing specific capacities. By employing a multi-faceted narrative assessment system, we can simultaneously explore AI-empowered chatbots’ proficiency to use prompts to asses global features of object relations and specific capacities simultaneously.

**Table 1:** Descriptions of the Scales for the Social Cognition and Object Relations Scales – Global Rating Method.

Scale		Brief description
Complexity of Representations for people	COM	Assesses differentiation and elaboration of internal features (e.g., affects; motives; thoughts). Lower scores indicate poor self-other differentiation or minimal discussion of internal states, while higher scores involve description of internal features, link internal features to characters’ actions, reactions, and perceptions, and evidence differentiation between characters.
Understanding Social Causality	SC	Assesses the extent to which events within a narrative are presented in organized and coherent sequence. Lower scores indicate less coherence and integration, and high scores indicate more.
Affective Quality of Representations	AFF	Assesses the emotional tone of the narrative. Lower scores indicate the narrative is communicated through the lens of negative emotions, while higher scores indicate more positive emotionality.
Emotional Investment in Relationships	EIR	Assesses the nature of interpersonal interactions and feelings toward others. Lower scores indicate negative interactions, self-centeredness, or limited relatedness, while higher scores indicate more positive interpersonal interactions, connections, and reciprocity.
Emotional Investment in Values and Moral Standards	EIM	Assesses if there is consideration of values and standards within the narrative. Lower scores indicate lack of standards, self-centeredness, or lack of remorse/empathy, while higher scores indicate focus on belief systems, consideration of responsibility, and use of standards to guide actions.
Experience and Management of Aggressive Impulses	AGG	Assesses capacity to experience and express anger in maladaptive to adaptive ways. Low scores indicate impulsive expressions of anger, inability to inhibit anger, or passive aggressive acts, while higher scores indicate capacity to inhibit anger, verbalization of anger, and efforts to resolve conflicts with others verbally.
Self-Esteem	SE	Assesses attitudes toward self. Lower scores indicate negative actions directed at the self, negative self-views, and sense of inadequacy, while higher scores indicate positive and realistic self-appraisals and sense of adequacy and worth.
Identity and Coherence of Self	ICS	Assesses degree of self-consistency and sense of identity. Lower scores indicate fragmented, dissociative, or unstable self-experiences, while higher scores indicate clear sense of purpose, commitment to goals, and capacity to navigate challenges during goal pursuit.

The SCORS-G is among the most widely studied narrative systems (for a detailed review, see Stein and Slavin-Mulford, 2018). However, it is resource-intensive and time-consuming to master. New raters undergo a rigorous 8-to-12-week training process. Training requires a detailed review of the SCORS-G manual (Stein et al., 2011; Stein and Slavin-Mulford, 2018), which provides chapters for each subscale that outline complex rules for assigning ratings. It also includes examples and rationales for why a given rating is assigned. The SCORS-G manual contains practice narratives rated by the master assessors to serve as a “gold standard” (i.e., ground truth ratings). These are used to evaluate whether new raters have achieved acceptable reliability.

Rater proficiency for the SCORS-G system differs based on a rater’s intended role. When ratings are made by teams (and the final rating is the average of all raters), average-rater ICCs (ICC [2, N]) of 0.70 or higher are sufficient to rate SCORS-G scales. However, when an individual rater will be the only one to provide ratings, they must achieve *single-rater* intraclass correlation coefficients (ICC [2, 1]) of 0.70 or higher. Achieving acceptable single-rater ICCs require notably higher levels of absolute agreement between raters and gold standards compared to average-rater ICCs, because the assumption is that the rater will be the only rater to make ratings. Given that clinicians often work independently and make ratings without other raters, meeting this higher standard is essential for ensuring accurate ratings.

The distinction between single-rater and average-rater reliability has implications for assessing chatbot performance. If chatbots achieve acceptable levels of single-rater reliability compared to gold standard ratings, it suggests they may be able to make reliable assessments independent of human involvement. This would indicate that the prompts effectively enable chatbots to rate narratives autonomously. Conversely, if chatbots do not meet single-rater reliability requirements but achieve acceptable average-rater reliability, this would still be beneficial. It would suggest that that AI chatbots can function as supplementary raters alongside a proficient human rater. Such an arrangement would still enhance the speed of narrative research by reducing the training burden on experts.

### 1.4 The present study

While narrative assessment has a long and rich history, its progress is hampered by the significant time, resource and training demands it places on researchers and clinicians. A review of the literature reveals that studies on self-report methods vastly outnumber those using performance-based assessments, like narrative analysis. AI has the potential to revolutionize narrative analysis, making it more efficient, less resource-intensive, and more accessible to practitioners and researchers. The purpose of the present study is to explore whether expert-crafted prompts can enable pretrained, general purpose AI models to reliably rate narratives using the SCORS-G. Additionally, we investigated whether expert-crafted prompts are more effective than basic prompting approaches. This study addresses the following research questions (RQs):

RQ1: Can experts develop and refine prompts that enable *Llama-2-70b* to reliably rate the SCORS-G?

RQ2: Will expert-refined prompts enable chatbots, that were not utilized in the prompt-refinement process, to reliably rate the SCORS-G?

RQ3: Will averaging ratings from two different AI chatbots prove more reliable ratings than relying on a single chatbot alone?

RQ4: Are detailed, expert-refined prompts superior to basic prompts with regards to reliability and level of absolute difference between chatbot ratings and gold standard ratings?

Beyond these specific research questions, we anticipated that chatbots would show different levels of proficiency across subscales. Thus, while we were primarily concerned with the SCORS-G global score to evaluate our three research questions, we also explore chatbots’ performance when rating SCORS-G subscales.

## 2 Methodology

**Figure 1:**
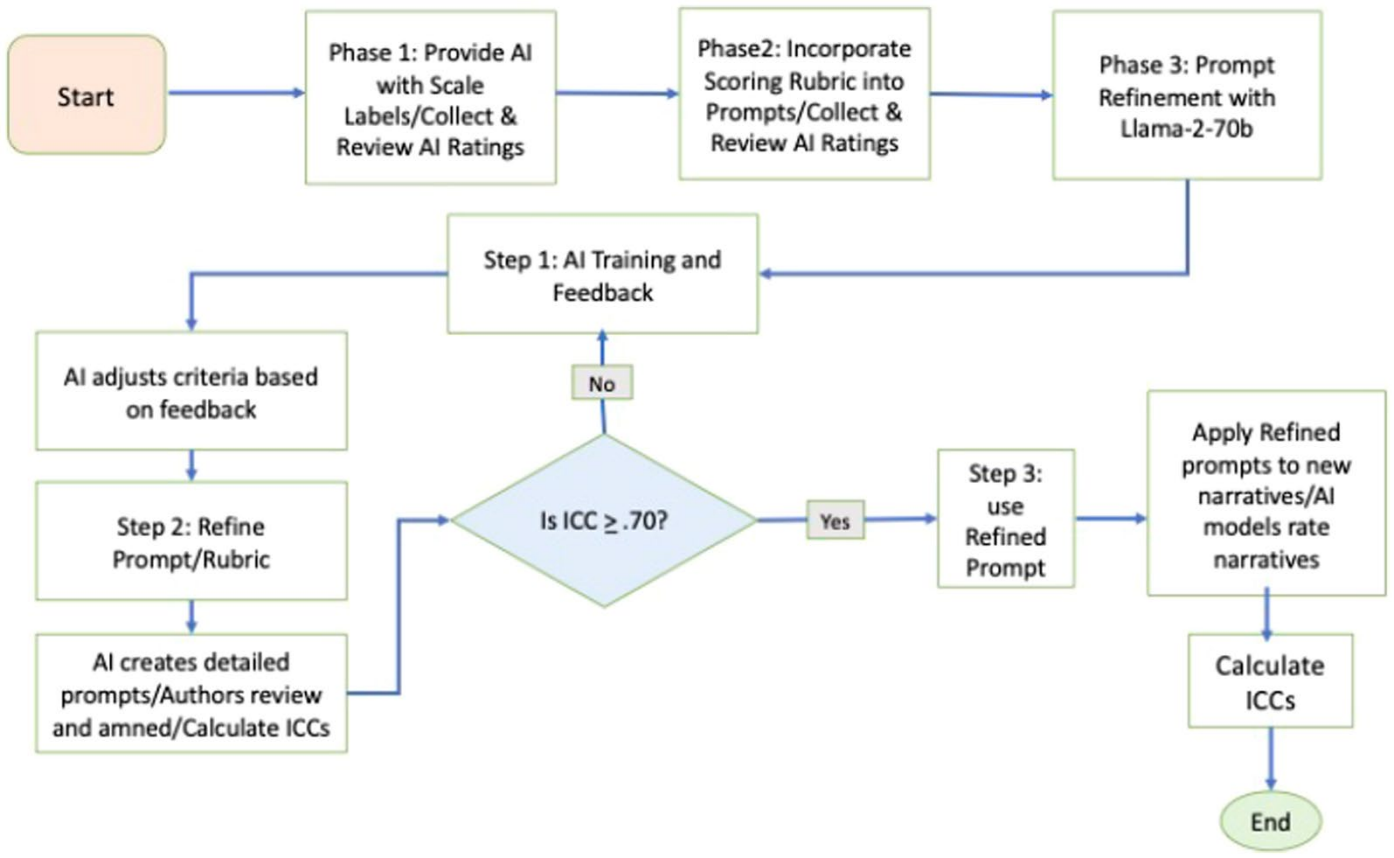
Flowchart of prompt refinement process.

To craft, refine, and assess our expert-refined prompts, we employed a systematic approach involving three phases. Figure 1 visualizes the steps involved in each phase of this process. In Phase 1, two experts (the authors) reviewed SCORS-G training materials to extract decision-making rules, which were then reworded to create the initial expert-crafted prompts. In Phase 2, these experts utilized *Llama-2-70b* (Meta, 2023) with the expert-generated prompts to rate practice narratives. They queried *Llama-2-70b*, refined the prompts based on the responses, and repeated this process until the prompts performed satisfactorily. In Phase 3, we assessed the utility of these expert-crafted, refined prompts by having two separate AI models—*ChatGPT-4* (OpenAI, 2023) and *CLAUDE-2-100k* (Anthropic, 2023), which were not involved in the prompt-refinement process—rate a larger set of narratives. We evaluated the reliability of these AI ratings against those provided by gold standard manual ratings and compared the performance of our expert-refined prompts to more basic prompts. The authors were not involved in creating the gold standard manual ratings which helps maintain the integrity of the process. We also explored whether averaging ratings across the two AI models yielded more reliable results than relying on a single AI. Throughout all phases, the authors interfaced with the AI models via the Platform for Open Exploration (POE), developed by Quora (2023), which provides access to several AI-empowered chatbots using advanced large language models.

### 2.1 Phase 1: expert-crafted prompt development

The goal of this phase of the study was to develop expert-crafted prompts that create a set of instructions emulating what human raters acquire from rigorous training. The experts who generated and refined prompts for this study were two licensed clinical psychologists, each with over 15 years of experience with the SCORS-G system. Both experts have achieved expert-level proficiency with the SCORS-G. One expert has trained 11 sets of human raters, consulted on the SCORS-G training manual, and co-edited a special section of the *Journal of Personality Assessment* dedicated to the SCORS-G. Both experts are university researchers, members of the Society for Personality Assessment, and engage in clinical personality assessment.

Experts followed recommendations for writing prompts such as writing prompts as instructions, using of simple language, using of affirmative language (rather than telling the chatbot “what not to do”), and writing prompts that informed the chatbot it should classify inputs (i.e., narratives) into one of seven category ratings (DAIR.AI, 2024). The experts also generated language to clarify how criteria for assigning ratings of 1–7. To develop this language, the experts conducted a detailed review of SCORS-G training manual (Stein and Slavin-Mulford, 2018) and extracted statements that inform scoring decisions. Such statements included descriptions of rating rules, rating rationales provided by mater assessors for the example narratives, and suggestions for avoiding common rating errors included in the manual. The experts identified redundancies to streamline the content and simplify instructions. Finally, they reworded statements into instructive prompts that could be given to the chatbots. These served as the expert-generated prompts. Example narratives and practice narratives in the SCORS-G manual are diverse, including narratives from early memories, stories generated to pictures, and a small number of psychotherapy segments. Thus, when generating prompts, the experts used a level of abstraction that sought to enable chatbots to rate different types of narratives (rather than using language specific to a particular narrative type).

### 2.2 Phase 2: developing expert-refined prompts

To refine the expert-crafted prompts, we employed *Llama-2-70b* (Meta, 2023). We first informed *Llama-2-70b* that we would be providing detailed instructions (the expert-crafted prompts) for rating a narrative. There is no identifying information on any of the narratives. After *Llama-2-70b* indicated its understanding, we provided a practice narrative and had *Llama-2-70b* make the rating. When the rating was correct, we informed *Llama-2-70b* it was correct and asked to explain its reasoning to better understand how it was using prompts. If the rating was inaccurate, we conducted an inquiry. Specifically, we asked *Llama-2-70b* to explain its initial rating, shared the correct rating with an explanation, and asked *Llama-2-70b* to make suggestions for refining prompts to improve accuracy. While considering *Llama-2-70b*’s feedback, the experts retained responsibility for modifying the prompts, drawing on their experience in training human raters.

Prompt-refinement was iterative. After the initial round, we initiated a new iteration with *Llama-2-70b*. We again informed it that we would provide it with detailed prompts for rating narratives and when it indicated understanding, we again provided it a practice narrative to rate. This time, we did not immediately engage in a query process. Instead, we repeated this process 10 times until *Llama-2-70b* had rated all 10 practice narratives. We next calculated average-rater intraclass correlation coefficients (ICCs [2, 2], two-way mixed-effects models, absolute agreement). Human raters must achieve an average-rater ICC (2, 2) of 0.70 or greater to demonstrate minimally sufficient proficiency. Thus, we set this value as our critical cutoff for concluding the expert-refinement process. When agreement fell below this threshold, we identified specific narratives that the AI rated poorly. We had *Llama-2-70b* re-rate these and then we queried as described in prior paragraph to clarify its rationales for ratings. The experts then further refined the prompts based on *Llama-2-70b*’s explanations and suggestions. This cycle continued until prompts achieved an average-rater ICC (2, 2) of 0.70 or higher for the subscale. We arbitrarily set 10 cycles as the max limit of iterations for achieving this aim, assuming that if reliability could not be generated in this number of cycles it would indicate that the chatbot was not sufficiently able to perform the task even with refinement.

### 2.3 Phase 3: evaluating the expert-refined prompts

The focus of Phase 3 was to evaluate the performance of our expert-crafted prompts relative to more basic types of prompts. To do this, we assembled a sample of 58 narratives that had gold standard ratings by SCORS-G master assessors. This dataset included 10 narratives per scale from the SCORS-G manual (Stein and Slavin-Mulford, 2018) and an additional 48 narratives sourced from other SCORS-G training materials (Stein et al., 2011). We included these additional 48 narratives to ensure that the prompts could accurately assess narratives beyond those used in the expert-refined prompt generation process. This resulted in a total of 58 narratives, per scale, with gold standard ratings. We provided *ChatGPT-4* (OpenAI, 2023) and *CLAUDE-2-100k* (Anthropic, 2023) with the expert-refined prompts and had it rate the 58 narratives for SCORS-G subscales. We calculated single-rater ICCs (2, 1) and average-rater ICCs (2, 2) for the ratings produced by *ChatGPT-4*, *CLAUDE-2-100k*, and the average of both chatbots. We followed recommendations by Koo and Li (2016) to characterize interrater reliability with ICCS: values lower than 0.50 are poor, values from 0.50 to 0.75 indicate moderate reliability, values between 0.75 and 0.90 indicate good reliability, and values of 0.90 or greater indicate excellent reliability.

We also compared expert-refined prompts to more basic prompts; specifically, vanilla prompts and one-shot prompts. Vanilla prompts are straightforward requests that provide minimal context and minimal instructions for completing a task. They require chatbots to rely almost exclusively on their pretraining (Wolfe, 2024). As such, they also provide a baseline assessment for a chatbot’s capacity to perform a task. All vanilla prompts used the following format: “I’m going to provide you with a narrative. You will rate the narrative on a scale of 1–7 regarding the ‘[name of the SCORS-G scale].’ A score of 7 indicates more positive and mature content, while a score of 1 reflects more negative or immature content.” The only variation among the eight prompts was the specific SCORS-G subscale name referenced in the prompt.

One-shot prompts provide an example or a limited amount of instructional information for completing a specific task (Zhang et al., 2023). The SCORS-G includes an anchor form providing a brief description of rating anchors for each subscale. It is intended for use by human raters that have completed training and are familiar with advanced decision-making rules. We used anchors from this form to create one-shot prompts for each subscale. For instance, for the Complexity of Representations of People scale, the one-shot prompt stated: “I’m going to provide you with a narrative. You will rate the narrative for Complexity of Representations of People on a scale of 1–7. 1 = egocentric, sometimes confusing thoughts, feelings, or attributes of the self and others; 3 = describes personalities and internal states in minimally elaborated, simplistic ways, or splits representations into good and bad; 5 = stereotypical or conventional representations, integrating both good and bad characteristics of self and others; 7 = psychologically minded, with insight into self and others, demonstrating considerable complexity.” The SCORS-G anchor form is included in Supplementary material.

## 3 Results

RQ1 asked if it was possible to generate expert-refined prompts that achieved minimally acceptable reliability estimates for the SCORS-G subscales. The expert-refined prompt generation process resulted in a series of prompts that enable *Llama-2-70b* to achieving average-rater ICCs (2, 2) of >0.70 for all subscales. No scale required more than 10 iterations to achieve this standard. Thus, we moved onto the next phase.

### 3.1 Interrater reliability of expert-refined prompts with *ChatGPT-4* and *CLAUDE-2-100k*

RQ2 focused on if expert-refined prompts empowered chatbots to rate narratives reliably. The columns in Table 2 labeled *ChatGPT-4* and *CLAUDE-2-100k* present single-rater ICC (2, 1) and average-rater ICC (2, 2) reliability estimates for each chatbot. Both chatbots just barely achieved the minimum required estimate of 0.70 for the global scale. These estimates were in the upper portion of the moderate interrater reliability range. Estimates for both chatbots were above the reliability threshold for average-rater ICCs (2, 2), indicating that they are likely to perform best when averaged with at least one human expert.

**Table 2:** Intraclass correlation coefficients assessing interrater agreement with gold standard ratings for the expert-refined prompts.

	*ChatGPT-4*		*CLAUDE-2-100k*		Average of Chatbots	
	Single	Average	Single	Average	Single	Average
Global	0.70	0.82	0.70	0.82	0.77	0.87
COM	0.73	0.85	0.60	0.75	0.72	0.85
SC	0.67	0.80	0.62	0.77	0.75	0.86
AFF	0.86	0.92	0.79	0.88	0.87	0.93
EIR	0.73	0.84	0.70	0.82	0.76	0.87
EIM	0.58	0.73	0.68	0.81	0.71	0.83
AGG	0.82	0.90	0.75	0.86	0.89	0.94
SE	0.57	0.72	0.80	0.89	0.76	0.87
ICS	0.64	0.78	0.68	0.81	0.72	0.83

Single = Interrater reliability estimates based on a single-rater, chatbot or human expert (ICC 2,1); Average = Interrater reliability estimates if ratings are averaged, chatbots and human expert (ICC 2, 2); Average of Chatbots = Ratings for the two Chatbots are averaged and treated as a single-rater.

Analysis of subscale estimates indicated that both *ChatGPT-4* and *CLAUDE-2-100k* were able to use expert-refined prompts to effectively rate AFF, EIR, and AGG. Each chatbot also evidenced strengths and weaknesses. Specifically, *ChatGPT-4* produced single-rater ICC (2, 1) estimates below the acceptable threshold for SE, while *CLAUDE-2-100k* performed admirably (single-rater ICC [2, 1] = 0.80). While both chatbots rated AFF at an acceptable level, *ChatGPT-4* (ICC [2, 1] = 0.86) proved a bit more reliable than did *CLAUDE-2-100k* (ICC [2, 1] = 0.79). This pattern aligns with expectations that differences in pretraining are likely to alter how specific AI models and chatbots interpret, assess, and rate narratives.

RQ3 asked if averaging ratings from two chatbots might mitigate these issues and prove superior to reliance on a single chatbot. Estimates for this approach are shown in Table 2 under the heading “average of chatbots.” As can be seen in Table 2, treating the average of the two chatbots as a single-rater was superior to using either *ChatGPT-4* or *CLAUDE-2-100k* alone. Reliability estimates for the global score, using this approach, increased from moderate (ICC (2, 1) = 0.70) to good (ICC (2, 1) = 0.77), with single-rater and average-rater ICCs that were larger than estimates based on an individual chatbots for all subscales. These data suggest that answer to RQ3 is “yes;” averaging ratings from two chatbots produced superior reliability relative to relying on a single chatbot. When an average of chatbots is used as a single-rater, the answer to RQ2 is also “yes.” Specifically, expert-refined prompts enable chatbots to reliably rate narratives on their own (i.e., single-rater). However, they are particularly adept when ratings are used in conjunction with at least one human expert (i.e., average-rater). Given these findings, we employed ratings based on the average of both chatbots to for all subsequent analyses assessing prompt types.

### 3.2 Comparing interrater reliability estimates across prompt types

RQ4 was concerned with determining if detailed, expert-refined prompts were superior to more basic forms of prompting. To address this question, we compared reliability estimates for vanilla prompts, one-shot prompts, and expert-refined prompts. Table 3 depicts the single-rater and average-rater ICCs by prompt type. Only the expert-refined prompts achieved an acceptable single-rater estimate (ICC [2, 1] = 0.77) in the “good” range for interrater reliability. The single-rater estimates for the global score for the vanilla prompts were poor (ICC [2, 1] = 0.48) and the one-shot prompts were in the lowest portion of the moderate range (ICC [2, 1] = 0.56).

**Table 3:** Comparison of ICCs for the global rating and subscales using averaged chatbot ratings across prompt types.

	Expert-refined prompts		Vanilla prompts		One-shot prompts	
	Single	Average	Single	Average	Single	Average
**Global**	**0.77**	**0.87**	**0.48**	**0.61**	**0.56**	**0.68**
COM	0.72	0.85	0.14	0.24	0.52	0.68
SC	0.75	0.86	0.13	0.24	0.13	0.22
AFF	0.87	0.93	0.87	0.93	0.80	0.89
EIR	0.76	0.87	0.66	0.80	0.79	0.88
EIM	0.71	0.83	0.49	0.66	0.60	0.75
AGG	0.89	0.94	0.39	0.56	0.30	0.44
SE	0.76	0.87	0.60	0.75	0.65	0.79
ICS	0.72	0.83	0.56	0.71	0.69	0.81

Global = the average ICC across the eight scales of the SCORS-G; Single = Interrater reliability estimates for a single-rater, human specialist, or AI (ICC 2,1); Average = Interrater reliability estimates for ratings based on multiple raters; human specialists and AI (ICC 2,2); AI Agreement = Interrater reliability for the average of two AIs (no specialists).

Only the expert-refined prompts produced acceptable single-rater ICCs for the eight subscales (i.e., ICCs [2, 2] ≥ 0.70). In contrast, the vanilla prompts only achieved an acceptable estimate for AFF (ICC [2, 1] = 0.87) and the one-shot prompts only produced acceptable estimates for AFF (ICC [2, 1] = 0.80) and EIR (ICC [2, 1] = 0.79). A notable implication of the estimates in Table 3 involves variability across subscales. While there was some variability for the expert-refined prompts, single-rater estimates were consistently in the upper portion of the moderate range or better (single-rater ICCs [2, 1] ranged from 0.71 to 0.89). By contrast, the vanilla and one-shot prompts single-rater ICCs (2, 1) below 0.50 for SC and AGG. Further, even when used in conjunction with a human expert, average-rater ICCs indicate were not in the acceptable range for the global scale several subscales. These data suggest it would be inadvisable to use the vanilla or one-shot prompts.

### 3.3 Comparing absolute differences across prompt types

To further assess RQ4, we experimentally compared average absolute differences between gold standard ratings and ratings produced by each type of prompt—expert-refined, vanilla, and one-shot. For example, if the chatbot average for a narrative was 4. and the gold standard rating was three, this would generate an absolute difference score of 1. If the chatbot average for a narrative was 1 and the gold standard rating was 5, this would generate an absolute difference score of 4. Thus, the lower the absolute difference score, the closer to gold standard ratings. Average absolute differences for each prompt type are shown in Table 4 in the columns marked expert-refined, vanilla prompt, and one-shot prompts.

**Table 4:** One-way ANOVA results comparing average absolute differences across the three prompt types.

	Mean absolute difference			*F*	*η* ^2^	LSD post-hoc tests
	One-shot	Vanilla prompt	Expert-refined			
Global	0.93	0.85	0.50	31.53**	0.27	Expert < Single Shot < Vanilla
COM	1.68	1.04	0.66	23.97**	0.22	Expert < Vanilla < Single Shot
SC	0.98	1.24	0.49	12.87**	0.13	Expert < Single Shot < Vanilla
AFF	1.47	0.36	0.47	52.21**	0.38	Expert & Vanilla < Single Shot
EIR	0.69	0.61	0.62	0.35	<0.01	None
EIM	0.43	0.79	0.43	7.37**	0.08	Expert & Single Shot < Vanilla
AGG	0.64	1.17	0.26	27.90**	0.25	Expert < Single Shot < Vanilla
ICS	0.73	0.84	0.51	4.31*	0.48	Expert < Single Shot & Vanilla
SE	0.84	0.76	0.53	4.06*	0.05	Expert < Single Shot & Vanilla

**p < 0.01; *p < 0.05; LSD, Least Significant Differences post-hoc tests; <, difference between absolute mean differences is statistically significant at the *p* < 0.05 level.

To statistically assess differences among prompt types, we used a one-way ANOVA, where the dependent variables were the SCORS-G global score and the eight subscales. As shown in Table 4, significant differences among the prompt types emerged for the global score, *F* (2, 171) = 31.53, *p* < 0.001; producing a large effect size (*η*^2^ = 0.27; Lakens, 2013). Least Significant Difference (LSD) post-hoc tests indicated that expert-refined prompts produced significantly lower discrepancies for the global score relative to the vanilla and one-shot prompts (which did not differ significantly from one another).

Regarding subscales, significant differences emerged across prompt types for AFF (*F* (2, 170) = 52.21, *p* < 0.001), AGG (*F* (2, 170) = 27.90, *p* < 0.001), COM (*F* (2, 170) = 23.97, *p* < 0.001), SC (*F* (2, 170) = 12.87, *p* < 0.001), EIM (*F* (2, 170) = 7.37, *p* < 0.001), ICS (*F* (2, 170) = 4.31, *p* = 0.02) and SE (*F* (2, 171) = 4.06, *p* = 0.02). No significant differences were observed for the EIR (*F* (2, 169) = 0.35, *p* = 0.70). Not only was the average absolute difference consistently lowest for the expert-refined prompts, LSD post-hoc tests indicated that expert-refined prompts had significantly lower absolute differences compared to the vanilla and one-shot prompts for COM, SC, SE, AGG and ICS. They also produced significantly lower absolute differences, relative to the vanilla prompts for EIM; and statistically lower absolute differences, relative to the one-shot prompts, for AFF. Notably, none of the prompt types differed for EIR. One implication of this is that both chatbot’s pretraining appears to enable them to rate this aspect of narratives well, with minimal need for input.

## 4 Discussion

The present study explored the potential of AI in assessing psychological narratives using the SCORS-G. Expert-refined prompts enable AI models, specifically *ChatGPT-4* and *CLAUDE-2-100k*, to rate narratives reliably and consistently with gold standard ratings from highly proficient human raters. Expert-refined prompts were particularly impressive at achieving acceptable reliability estimates when final ratings were generated by averaging ratings across two AI-empowered chatbots. Findings provide a proof of concept for use of AI in narrative assessment. AI has potential to render research in this area more efficient, less resource-intensive, and more broadly accessible (Koutsouleris et al., 2022; Khurana et al., 2023).

If expert-refined prompts could be effectively generated and refined for use with the SCORS-G, it is quite possible that prompts could be developed to assist researchers and clinicians using other narrative-rated systems. The implications of automation in narrative assessment could be profound. Ideally, personality assessment and research make use of multiple methods (Bornstein, 2014). However, research into narrative-based assessment is much smaller than the research base for self-report tools, which has limited use of these assessment techniques in practice. This discrepancy between literatures is driven, in large part, by the fact that self-report research far faster to conduct. Narrative rating methods require considerable time, resources, and efforts. Rating even modest number of narratives in a study takes several months to years. Development of reliable and accurate AI models or prompts to automate narrative ratings (or simply increase efficiency) could enable researchers to score narratives as quickly as they do with self-report measures. Increasing the research base in this area would empower researchers and clinicians to use multiple methods, which is considered best practice in the field of personality assessment (Hopwood and Bornstein, 2014). While this study alone cannot achieve this aim, it serves as an important initial step in this direction.

### 4.1 Implications for narrative ratings with the SCORS-G

Findings are particularly relevant for those using the SCORS-G. Expert-refined prompts produced stronger reliability estimates and less absolute discrepancy with gold standard ratings relative to other prompt types. This was particularly true at the global level, though examining findings at the subscale level provides a more nuanced picture emerges. The expert-refined prompts were notably superior in assessing cognitive dimensions of object relations (i.e., COM and SC) and in evaluating regulation of anger and aggression (i.e., AGG). They were also moderately superior for assessing use of internal standards to regulate impulses (EIM) and in assessing self-evaluation (SE), but distinctions were smaller. An implication of this is that the greater level of detail included in the expert-refined prompts is necessary for chatbots to assess the most complex and abstract aspects of object relations. In contrast, all three prompt types effectively assessed the emotional dimensions of narratives. An implication of this is that chatbot pretraining may enable them to effectively rate the affective quality of representations in narratives with minimal input. During training, human raters typically achieve acceptable levels of reliability faster on for the AFF subscales as well. In summary, while expert-refined prompts were superior overall—particularly for key subscales—the inherent abilities of chatbots to accurately rate emotional and relational aspects of narratives is impressive.

Findings also have implication for the use of chatbots in automating or assisting in assigning SCORS-G ratings. When the average of two chatbots using expert-refined prompts were used, single-rater ICCs exceeded acceptable thresholds for both single-rater and average-rater ICCs for the global scale and all subscales, suggesting that AI-empowered chatbots may be able to use these prompts to rate narratives without the need for human experts. While estimates were in a range that would support the use of expert-refined prompts without human raters, we encourage psychologists to be cautious when considering this option, as this is the first study on this topic. Additional research replicating these findings with other samples of narratives that have been rated by highly expert raters are necessary to ensure generalizability of the present findings. More research is necessary before AI is used to automate SCOR-G ratings, especially in clinical settings.

The expert-refined prompts may still have immediate, practical use. Average-rater ICC estimates were consistently strong, indicating that the current expert-related prompts may be helpful as supplemental raters working in conjunction with human raters. Researchers could use chatbots to increase the number of available raters in a study. Similarly, chatbot raters could be used to identify “rater drift” (i.e., a rater failing to assign the rating they would typically give due to a state effect, such as fatigue, hunger, or simply making several ratings in a single rating episode). For example, when chatbots, using expert-refined prompts, produce ratings that differ from a human rater at a level of two or more, these narratives could be flagged for review by human raters to determine if a mistake was made. This approach may be particularly useful for those using the SCORS-G in clinical settings (where the clinician is typically the only person making the ratings) by serving as a “check-and-balance” that could quickly help the rater identify cases of drift. Additionally, chatbot models may be useful for rater training. In our work, when we asked the AI to explain the ratings it made, it provided compelling arguments that were like rationales from human experts. While it is unlikely chatbots could be involved in SCORS-G training currently, present findings show potential for the future.

### 4.2 Implications for developing expert-refined prompts for other narrative systems

Some aspects of the study extend beyond the SCORS-G. While many of these may be obvious to engineers who are well-versed in AI research practices, they may be of interest to other psychological researchers hoping to work with AI. The study highlights the importance of experts in the prompt-refinement process. Development of expert-refined prompts can optimize AI-empowered chatbot’s capabilities for psychological research without necessitating direct manipulation of underlying algorithms. Prompts are relatively easy to deploy, which could simplify the use of AI and broadens its applicability in psychological settings (Bender et al., 2021; Gao et al., 2019; Liu et al., 2021). In our study, expert-refined prompts, developed through an iterative process, were reliable for making complex ratings. Further, they proved superior to vanilla and one-shot prompts in several ways (Zhang et al., 2023). This finding underscores the significance of domain expertise in developing effective prompts for complex tasks (Priyadarshana et al., 2024).

**Figure 2:**
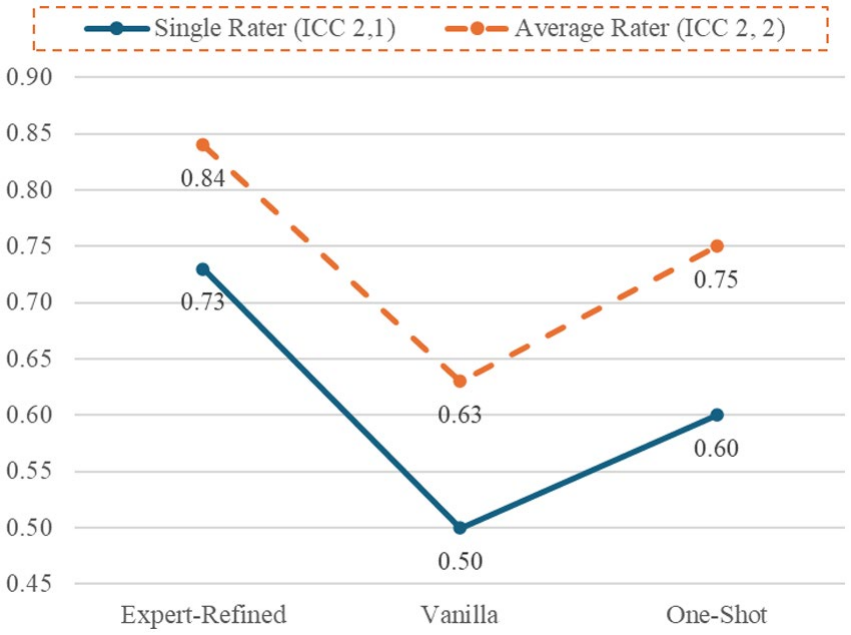
Comparison of single-rater and average-rater ICCs for the SCORS-G Global Rating expert-refined prompts, vanilla prompts, and one-shot prompts.

The study also provides a model for generating and assessing expert-refined prompts to enable chatbots to rate narratives. The SCORS-G system contains several subscales that differ considerably in the content they assess. If expert-refined prompts were useful to these diverse subscales, it seems likely that expert-refined prompts could be generated for other well-validated systems for assessing agency and communion, motivation, adult attachment, mentalization, and so on. We recommend researchers follow the prompt refinement process, as outlined in our flowchart (Figure 1). This approach may be useful in generating and refining prompts for a wide range of narrative assessment systems. We advise using one chatbot in an iterative prompt-refinement process and then studying prompt effectiveness using chatbots that were not involved in prompt-refinement. By using chatbots independent from the one used for prompt refinement, the researcher can assess the quality of the prompts themselves (separate from the chatbot they were originally developed with). When independent chatbots can use prompts to rate new narratives (i.e., narratives that were not used during the prompt refinement process), this supports the utility of the prompts themselves. Inclusion of more basic prompts, such as vanilla and one-shot prompts, is also advised as these are useful for estimating chatbots’ baseline proficiency for assessing various constructs and providing comparison standards for expert-refined prompts (Figure 2).

It is common practice to rely on a single AI LLM for such tasks. For instance, van Genugten and Schacter (2024) used a single LLM to rate narratives from the Autobiographical Interview. While this approach is not flawed, our study highlights the value of treating employing multiple chatbots or AI models as independent raters. When tasks require a high level of sophistication, employing multiple chatbots offer advantages over relying on a single model. AI models and chatbots may exhibit biases, based on pretraining, impacting how they interpret prompts, draw inferences, and assign ratings. To illustrate this point, in this study, neither chatbot achieved acceptable single-rater estimates for SC, EIM, or ICS. However, averaging ratings across chatbots into a single-rater resulted in acceptable reliability estimates for all subscales including SC, EIM, and ICS *where neither chatbot was able to reliably rate the subscale.* Just as humans differ in the personal experiences they bring to training, so too are chatbots and AI models impacted by the specific datasets and training techniques used to create them. Averaging across chatbots with different pretraining may reduce bias associated with any one specific chatbot. This supports the use of multiple chatbots or AI models to make complex ratings. Averaging their scores could mitigate risks associated with reliance on a single chatbot or AI model.

### 4.3 Limitations

While our findings are encouraging, it is essential to recognize the limitations of this study. First, given the intensive process and multiple iteration nature of the expert-refinement process for the prompts, we did not investigate how each change impacted overall reliability estimates (as this would have been overly burdensome and resulted in hundreds of individual analyses), opting instead to compare the final product of the expert-refinement process to more basic prompts. Second, we did not engage in any exploration of conditional factors (e.g., narrative type). Because the SCORS-G is used to rate an array of narratives, the training materials contains multiple narrative types (e.g., stories generated to pictures, segments of psychotherapy transcripts, early memories). As such, our sample of practice narrative contained multiple narrative types. Prompts were then tested using a larger sample of narratives that primarily included early memories. It is possible that prompt performance may have been higher if only early memories were used to create and refine prompts. In the future, researchers should investigate if conditional prompts (i.e., prompts intended for use with specific types of narratives) prove superior to more generalized prompts, such as the ones developed here. Third, because our narrative sample used to assess prompt quality was primarily composed of early memories, we did not possess enough narratives across narrative type to engage in a systematic comparison of how type impacted rating.

While not a limitation per se, this study did not use machine learning or fine-tuning procedures. Research using machine learning procedures on downloadable, adaptable large language models (LLMs) is needed to clarify their capacity to accurately rate narratives. The performance of these models could be compared with the pretrained chatbots, *ChatGPT-4* or *CLAUDE-100-K*, used in this study. If adaptable LLMs are found to be equal or superior to chatbots in terms of performance, this could offer significant advantages in terms of security and privacy, as users would be able to maintain control over their data and avoid relying on cloud-based services. Specifically, our research was confined to a narrow set of narratives and a single narrative assessment tool, the SCORS-G. To further validate the effectiveness of expert-refined prompts, future studies should investigate their application to other narrative assessment tools and explore the potential of AI in various domains of psychological research. Moreover, future research would benefit from rating a larger set of narratives, encompassing a broader range of content, language usage, and other factors that may impact the consistency and accuracy of expert-refined prompts. This would provide a more comprehensive understanding of the strengths and limitations of this approach.

Our study did not address the important ethical considerations surrounding the use of AI in psychological assessments. As highlighted by previous research (Asan et al., 2020; Al Kuwaiti et al., 2023), the integration of AI in psychological assessments raises significant ethical concerns that warrant further examination. Future studies should prioritize exploring these issues to ensure that the development and implementation of AI-based assessment tools are guided by a robust ethical framework. AI has the potential to complement and enhance human expertise, allowing for more efficient and accurate analysis of large datasets. However, this integration also necessitates collaboration between psychologists and AI experts to ensure the development of reliable and valid AI-based tools for psychological research. As the field progresses, it is crucial to address the ethical implications of AI in psychology, including issues of transparency, accountability, and fairness (Asan et al., 2020; Al Kuwaiti et al., 2023).

## Data Availability

The raw data supporting the conclusions of this article will be made available by the authors without undue reservation.
