# Supplementary material for: From Llama to language: prompt-engineering allows general-purpose artificial intelligence to rate narratives like expert psychologists
Source: Front Artif Intell. 2025 Feb 6;8:1398885. doi: 10.3389/frai.2025.1398885 (PMC11839667; doi:10.3389/frai.2025.1398885)
Supplement: Supplementary file 1 [file Data_Sheet_1.PDF]

SOCIAL COGNITION AND OBJECT RELATIONS SCALE
RATING FORM

Please rate the patient on each of the following dimensions, using the 1-7 scales indicated. Each scale is on a continuum, with higher scores indicating more mature or healthy functioning.

Complexity of representations of people: 1 = tends to be grossly egocentric, or to confuse his/her own thoughts, feelings, or attributes with others'; 3 = views the self and others with little subtlety or complexity; descriptions of people tend to be sparse, simple, one-dimensional, poorly integrated, or split into all-good or all-bad (e.g., tends to describe people as “nice,” “mean,” etc.); 5 = views of the self and others have some depth and complexity but are relatively conventional; is able to see people's strengths as well as weaknesses, and to take others' perspective; 7 = is psychologically minded; views of people are subtle, rich, and complex.

1 —— 2 —— 3 —— 4 —— 5 —— 6 —— 7

Affective quality of representations: (what the person expects from, and experiences in, relationships): 1 = tends to have malevolent expectations of relationships; often experiences people as abusive or intentionally destructive; 3 = tends to experience relationships as somewhat unpleasant, hostile, or indifferent, or to feel very alone; 5 = expectations of relationships are affectively mixed; tends to describe both positive and negative relationship experiences; 7 = has genuinely positive expectations of relationships, but is not "pollyannish" (i.e., can see people for what they are). Note: Where affective quality of representations of relationships tends to be bland, absent, limited, or defensively positive, code "4."

1 —— 2 —— 3 —— 4 —— 5 —— 6 —— 7

Capacity for emotional investment in relationships: 1 = tends to focus primarily on his/her own needs in relationships; to have unstable, tumultuous relationships; or to have few if any relationships; 3 = relationships tend to be shallow, lacking in depth, or based primarily on mutual participation in shared activity or mutual self-interest; 5 = demonstrates conventional sentiments of friendship, caring, love, and empathy in relationships; 7 = tends to have deep, committed relationships characterized by mutual sharing, emotional intimacy, interdependence, respect, and appreciation.

1 —— 2 —— 3 —— 4 —— 5 —— 6 —— 7

Emotional investment in values and moral standards: 1 = evidences a relative absence of moral values and concerns for the needs of others; may behave in selfish, inconsiderate, self-indulgent, or aggressive ways with little sense of remorse or guilt; 3 = shows signs of *some* internalization of standards (e.g., avoids doing “bad” things because knows others will think badly of him/her; thinks in relatively simple or childlike ways about right and wrong") but lacks mature feelings of guilt or remorse for wrongdoing and a capacity to override own desires that regulate behavior; 5 = is invested in moral values and experiences guilt for hurting other people or failing to meet moral standards; has conventional moral views; 7 = thinks about moral questions in a way that combines abstract thought, a willingness to challenge or question convention, and genuine compassion and thoughtfulness in actions. Note: Where the person is morally harsh and rigid toward self or others, code "4."

1 —— 2 —— 3 —— 4 —— 5 —— 6 —— 7

Understanding of social causality *(ability to understand why people do what they do)*: 1 = explanations of people's behavior or narrative accounts of interpersonal experiences tend to be confused, confusing, distorted, extremely sparse, or difficult to follow; "stories" of events tend to lack coherence; 3 = explanations of people's behavior or narrative accounts of interpersonal events tend to be slightly confusing; descriptions of interpersonal events often have incongruities that require "work" to understand fully; 5 = tends to provide straightforward narrative accounts of interpersonal events in which people's actions result from the way they experience or interpret situations; 7 = tends to provide rich, coherent, and accurate accounts of interpersonal events. Note: where the person tends to describe interpersonal events as if they "just happen," with little sense of why people behave the way they do (i.e., *alogical* rather than *illogical* narratives, which seem to lack any causal understanding), rate "2."

1 —— 2 —— 3 —— 4 —— 5 —— 6 —— 7

Experience and management of aggressive impulses: 1 = is physically assaultive, destructive, sadistic, or in poor control of aggressive impulses; 3 = tends to be angry, passive-aggressive, denigrating of others, physically abusive to self, or unable to protect self from escapable abuse; 5 = avoids dealing with anger by denying it, defending against it, or avoiding confrontations; 7 = can express anger and aggression and assert him/herself appropriately.

1 —— 2 —— 3 —— 4 —— 5 —— 6 —— 7

Self-esteem: 1 = views self as loathsome, evil, rotten, contaminating, or globally bad; 3 = has low self-esteem (e.g., feels inadequate, inferior, self-critical, etc.); 5 = displays a range of positive and negative feelings toward the self; 7 = tends to have realistically positive feelings about him/herself. Note: where person is grandiose, or alternates between overvaluation and devaluation of self, rate "4."

1 —— 2 —— 3 —— 4 —— 5 —— 6 —— 7

Identity and coherence of self: 1 = has multiple personalities; 3 = views of, or feelings about, the self fluctuate widely or unpredictably; lacks stable goals, ambitions, or core values; has an unstable sense of self; feels as if s/he "doesn't know who s/he is"; 5 = identity and self-definition are not a major concern or preoccupation; 7 = feels like an integrated person, with stable commitments to long-term ambitions, goals, values, and relationships.

1 —— 2 —— 3 —— 4 —— 5 —— 6 —— 7
