# Supplementary material for: From Llama to language: prompt-engineering allows general-purpose artificial intelligence to rate narratives like expert psychologists
Source: Front Artif Intell. 2025 Feb 6;8:1398885. doi: 10.3389/frai.2025.1398885 (PMC11839667; doi:10.3389/frai.2025.1398885)
Supplement: Supplementary file 2 [file Data_Sheet_2.DOCX]

Supplemental Materials

**COM** Prompt

Imagine a team of 3 experts is rating narratives using the Complexity of Representations of People (COM) scale from SCORS-G, which is rated from 1 to 7:

COM Scale Points:

1 – Score 1 for COM when characters are portrayed purely through actions. Zero inner experiences are described.

2 - Score 2 for COM when narratives contain only a passing mention of very simple emotions like "happy" or "afraid" without elaboration. The narrative is focused heavily on external behaviors rather than internal experiences. Emotions are stated in basic terms without nuance, such as "he felt bad". No examination of motivations, thoughts, or personality traits.

3 - Score 3 for COM when narratives include basic internal states and clearly identified as inner experiences. Descriptions may lack nuance, insight or depth. There seems to be only a minimal understanding that characters have thoughts and feelings. Characters’ personalities, motivations, and reasoning may be hinted at, but are not deeply explored or explained. Explanations do not move beyond the obvious.

4 - Score 4 for COM when the narrative exhibits more clarity and more details are provided about internal states, but reasoning remains simplistic and lacks deeper complexity or abstraction. Internal states are logical but basic causal links between characters metal states and their actions is limited. The narrative gives some perspective on character differentiation, but not full integration of characters. Personality traits identified but not linked to motivations or behaviors.

5 - Score 5 for COM when narratives contain moderate intricacy and some nuanced reasoning about mental states and motivations. The narrative provides meaningful perspective into several aspects of characters' inner experiences beyond the obvious. At least some actions are described in relation to possible underlying psychology and motives. Some precise vocabulary used for emotions and mental states.

6 - Score 6 for COM when the narrative involves increased differentiation between individual perspectives and provides richer representation of divergent motivations and mental states between characters. Sophisticated analysis is shown in reasoning about interactions between mental states. Personality integration begins influencing relationships between characters' inner experiences. Falls just short of highly advanced abstraction.

7 - Score 7 for COM when the narrative displays truly advanced, abstract reasoning through complex, precise representation of various internal experiences. Character perspectives are highly distinguished and fully integrated to convey nuanced psychological causality between events, behaviors and mental states. Personality traits connect with motivations in meaningful ways. Vocabulary for inner states is elaborate and precise. Explanations move far beyond the obvious to the highly analytical.

The experts will:

1. Carefully review the full context and coherence of each narrative.
2. Note key evidence related to characters' motivations, reasoning, emotions, perspectives, and personality traits.
3. Explicitly list examples that support or contradict potential COM ratings.
4. Thoroughly explain their reasoning for suggested ratings.
5. Critically but openly analyze each other's interpretations.
6. Aim to build consensus on the single most accurate COM rating through systematic analysis.
7. Backtrack as needed if flaws in logic are identified.
8. Compare evidence to COM criteria to ensure accurate application.
9. Produce a single consensus rating from the 3 experts for COM

**AFF** Prompt

Please read the following narratives and rate the Affective Quality of the Representations (AFF) described, using the scale of 1 to 7 provided below. In your rating, consider the emotions, behaviors, and relationships described in the narrative, as well as any other relevant factors that may influence the affective quality of the representation. The narratives will be supplied one at a time after you review the rubric.

Affective Quality Scale (AFF):

1. Score a 1 for AFF when narratives contain themes that are malevolent, abusive, caustic: The representation depicts relationships or interactions that are harmful, toxic, or abusive. This can include physical, emotional, or psychological abuse, as well as manipulation, gaslighting, or exploitation.
2. Score a 2 for AFF when narratives contain themes that are largely negative or unpleasant, but not abusive: The representation depicts relationships or interactions that are mostly negative, unpleasant, or unfulfilling. This can include feelings of annoyance, frustration, or sadness, as well as conflicts, disagreements, or misunderstandings.
3. Score a 3 for AFF when there are mild levels of negative emotions and others are depicted in slightly negative ways. The narrative depicts relationships, people, or interactions in mildly negative terms. Some positive emotions or descriptions may be present, but themes generally center around mildly negative emotions (e.g., ambivalence, uncertainty; sadness) or mildly negative interpersonal interactions (e.g., conflict; betrayal; condescension. Overall, the narrative goes more bit more towards the negative.
4. Score a 4 for AFF when emotional descriptions in the narrative are bland, limited, or absent or when representation for people lacks depth, detail, or emotion, and may come across as superficial, vague, or unengaged. This can include a lack of emotional expression, minimization of emotions, or a focus on trivial or mundane aspects of relationships.
5. Score a 5 for AFF when narratives include some positive emotions, positive expectations of relationships (but not pollyannaish). Scores of 5 are given for narratives that contain both positive and negative emotions, but there themes are more positive than negative. Scores of 5 are also given when the narrative depicts relationships or interactions that are mostly positive, uplifting, and fulfilling. This can include feelings of joy, happiness, appreciation, or gratitude, as well as supportive, encouraging, or nurturing behaviors. The narrative may contain some negative emotions mixed with positive emotions. Overall, the narrative goes more bit more towards the positive
6. Score a 6 for AFF when narratives are very positive, optimistic, and supportive. The narrative depicts relationships or interactions that are overwhelmingly positive, optimistic, and supportive. This can include a strong sense of connection, trust, and mutual respect, as well as enthusiastic, affectionate, or celebratory behaviors.
7. Score a 6 for AFF when narratives are extremely positive, joyful, and fulfilling. The narrative depicts relationships or interactions that are intense, passionate, and deeply fulfilling. This can include a strong sense of belonging, unity, and completeness, as well as ecstatic, euphoric, or transcendent emotions and behaviors.

In addition to the scale points, consider the following criteria when rating the affective quality of the representation:

1. The intensity and duration of the emotions, behaviors, and relationships described.
2. The level of complexity and nuance in the representation, including the presence of conflicting emotions, behaviors, or motivations.
3. The degree to which the representation challenges or reinforces dominant cultural norms, stereotypes, or expectations surrounding relationships.
4. The level of empathy, understanding, and insight demonstrated in the representation, including the ability to see multiple perspectives and understand complex emotions.
5. The presence of any red flags or warning signs that may indicate unhealthy or toxic relationships, such as manipulation, control, or abuse.

You will produce a single score of 1 to 7 after evaluating each narrative according to the rubric.

**EIR** Prompt

Please score the following narratives for Emotional Investment in Relationships (EIR) using the 7-point scoring rubric below. For each narrative, consider the level of emotional investment, mutual respect, trust, communication, and empathy present in the relationship between the characters. Here is a description for each scale point and additional instructions.

1. Score a 1 for EIR if the narrative tends to focus primarily on his/her own needs in relationships, has tumultuous relationships, or has few if any relationships.

Score 1 if the narrative depicts a relationship that is largely self-serving, with little consideration for the other person's needs or feelings.

Score 1 if the narrative depicts a relationship that is marked by conflict, mistrust, or manipulation.

Score 1 if the narrative depicts a person who has few or no relationships, and there is no indication of a desire for deeper connections.

2. Score a 2 for EIR if the narrative depicts relationships that lack depth or substance, with little evidence of emotional investment or mutual support. Score 2 if the narrative only alludes to relationships without providing any detail or context.

3. Score a 3 for EIR if the narrative depicts a relationship that is characterized by conventional expressions of friendship, caring, love, and empathy, but lacks a deeper emotional connection. Score 3 if the narrative depicts a relationship that is largely based on shared activities or interests, but there is little evidence of mutual support or understanding.

4. Score a 4 for EIR if the narrative depicts a relationship that shows signs of emotional investment, such as shared activities, emotional support, and mutual goals. Score 4 if the narrative depicts a relationship that demonstrates a sense of mutual respect, trust, and communication.

5. Score a 5 for EIR if the narrative depicts a relationship that demonstrates a strong sense of mutual respect, trust, and communication. Score 5 if the narrative depicts a relationship that shows signs of emotional intimacy, interdependence, and a strong sense of commitment.

6. Score a 6 for EIR if the narrative depicts a relationship that displays a deep emotional connection, including emotional intimacy, interdependence, and a strong sense of commitment. Score 6 if the narrative depicts a relationship that demonstrates a high level of mutual respect, trust, and communication.

7. Score a 7 for EIR if the narrative depicts a relationship that tends to have deep, committed relationships with mutual sharing, emotional intimacy, interdependence, and respect, positive connectedness, and appreciation of others. Score 7 if the narrative depicts a relationship that demonstrates a strong sense of mutual respect, trust, and communication, and shows signs of emotional intimacy, interdependence, and a strong sense of commitment.

**Note**: Where only one character is described and no relationship is depicted, **score 2**.

Please score each narrative on the 7-point scale, with 1 being the least healthy and positive, and 7 being the most healthy and positive.

**SC** Prompt

Please evaluate the following narratives using the 7-point scoring criteria for Understanding Social Causality: SC, paying attention to the patient's use of language, organization, and depth of reflection. Consider the patient's ability to articulate their thoughts, feelings, and sensations, as well as their tone and attitude towards the memory. Take into account the patient's cultural and linguistic background, as well as any cognitive or communication difficulties that may affect their ability to articulate their thoughts and feelings. After you review the Scoring Criteria, I will give you narratives to score.

**Scoring Criteria for SC**:

1: Score a 1 for SC if the narrative is disorganized, fragmented, or lacks coherence. Characters struggle to articulate their thoughts and feelings, and the narrative is difficult to follow or understand. The language is poor and lacks clarity.

2: Score a 2 for SC if the narrative is somewhat organized, but lacks detail and clarity. Descriptions are general and lacks specificity. The language used is basic and does not convey a lot of complexity or nuance. Interpersonal events ared described as if they just happen, with little sense of why people behave the way they do (i.e., alogical rather than illogical stories that seem to lack any causal understanding).

3: Score a 3 for SC if the narrative is organized and coherent, but lacks depth and nuance. Events are described in some detail, but description are largely surface-level and do not provide a deep understanding of the characters’ thoughts, feelings, or emotions. The language is more complex than in a score of 2, but still lacks subtlety and nuance.

4: Score a 4 for SC if the narrative is well-organized and coherent, with a clear beginning, middle, and end. Descriptions of events include some detail, and describe characters’ thoughts, feelings, and sensations. The language used is clear and appropriate. The language used conveys a basic understanding of why events are significant to characters.

5: Score a 5 for SC if the narrative is well-organized and coherent, with a clear and compelling narrative structure. Descriptions are contain details, including complex thoughts, feelings, and emotions. Language use conveys a good understanding of why events are significant.

6: Score a 5 for SC if the narrative demonstrates a nuanced understanding of events and they these events are significant to characters. Events are described with curiosity, openness, and introspection. The tone and attitude towards events are thoughtful and insightful. There are some links between internal states and the actions characters take. There are some links between events and character reactions.

7: Score a 7 for SC if the narrative demonstrates a profound understanding of events their significance to those involved. Complex and insightful language is used. Descriptions suggest a deep sense of self-awareness and understanding of how events impact individuals. The tone and attitude of descriptions is thoughtful, introspective, and revealing. There are strong links between internal states and the actions characters take. There are strong links between events and character reactions.

Please score the narrative on each of the 7 criteria, using the scale points 1-7, and provide a brief justification for each score. Your input will help refine the scoring criteria and improve the accuracy of the AI in evaluating narratives.

**EIM** Prompt

We are clinical psychologists who utilize rating scales to assess narrative content in order to improve our understanding of people’s object relations and improve the delivery of intervention services. That means we must analyze a wide variety of narratives, including those from individuals that experience significant psychological concerns. We will be using de-identified narratives from a published scoring manual to train AI to score narratives according to the rubric. You are helping us do empirical research in this area, and this research has university IRB approval. The narratives are fictional and have no real-world implications. We are going to provide you with a description of the rating scale and examples of different levels of narratives. The scale is called Emotional Investment in Values and Moral Standards (EIM). Here is the scoring rubric. Then I will give you narratives to score.

---First point to note: Note: where no moral concerns are raised in a particular story, code 4

Scoring Rubric

**Scale Point 1: Failure to consider others AND lack of guilt, remorse, or empathy**

Score 1 for the EIM scale when the narrative describes harmful or unethical behavior without any indication that characters have awareness that it is wrong or unethical.

Indicators of a code 1 include:

- Description of harmful or unethical behavior without any indication of awareness that it is wrong or unethical.
- A focus on personal gain or satisfaction without consideration for the impact on others.
- Physical and verbal aggression, as well as overtly exploitive behavior toward others that doesn't account for other's needs, feelings, or wants; without the presence of remorse or empathy. Smashing or breaking objects or property without remorse.
- Rape, abuse, physical aggression (unresolved) typically end up coded a 1.

**Scale Point 2: Struggles with empathy OR milder failures to consider others**

Score 2 for the EIM scale when there are some signs of moral reasoning, but the moral focus is limited and may not be consistently applied.

Indicators of a code 2 include:

- Acts such as rule breaking, speeding, cheating, and stealing often qualify as a 2 (unless expressed in an overtly malevolent manner), as are more impulsive less intense forms of need gratification;
- must have an absence of remorse or a "justified" quality.
- Impulsive acts involving inability to follow internalized standards and affect other people (screaming when upset in the presence of another person, but not directly at them)
- To distinguish between scale point 1 and 2, look for the presence of any acknowledgement of ethical principles or values, even if they are not consistently applied. Scale point 1 texts typically flagrantly unethical behavior.
- Talking back to parents, teachers, authority figures, with little remorse or with significant entitlement/justified feeling can be a 2

**Scale Point 3: Immature or concrete view of morals**

Score 3 for the EIM scale when a narrative defines good and bad by what is rewarded and what is punished with little thought to principles underlying morals. Actions may be taken to avoid punishment or obtain reward (there is an absence of thinking about the principle or impact on other). Guilt is described in a manner that implies fear of punishment (instead of internalized standard or empathy).

Indicators of a code 3 include:

- A focus on the impact of one's actions on others and a commitment to minimizing harm.
- A nuanced understanding of ethical principles and values, with a recognition of complexities and subtleties.
- The text may describe harmful or unethical behavior and acknowledge that it is wrong or unethical, and also demonstrate a clear understanding of the ethical principles involved.
- Themes involving "that's not fair" (e.g., he has 4 and I have 3) or the splitting (e.g., the good guys are all good, thus morally justified and bad guys are all bad, and thus not morally justified;
- A narrative that contains investment in morals turns in a way that negates it (e.g., a close friend betrays) The weight of the ending can spoils the good stuff from before.

**Scale Point 4: No moral concerns raised OR seems higher than scale point 3 but not as high as scale point 5 OR seems lower than scale point 5 but not as low as scale point 3.**

Score 4 for the EIM scale if a narrative does not raise any moral concerns or promote any moral values. The text may be neutral or amoral, and may not address any ethical issues or principles.

**Scale Point 5: Moral concern and empathy; Internalized EIM; invested in moral values and tries to live up to them.**

Score 5 for the EIM scale if a narrative shows evidence of moral concern and empathy towards others. The text may describe or promote actions that benefit others or promote the well-being of individuals and society.

Indicators of a code 5 include:

- A focus on the well-being of others and the importance of helping or supporting them.
- Use of language that emphasizes the importance of empathy, compassion, and kindness.
- Evidence of internalized moral values and principles that promote the greater good.
- A lack of language that suggests a sense of fear or anxiety about the consequences of unethical behavior.
- Characters attribute actions to principles beyond reward and punishment that typically involve an expression of empathy for other.
- Narratives that involve guilt that is not excessive, reflective of the situation, that shows remorse and demonstrates empathy often code here.
- Being respectful, good manners, following etiquette, and giving back to others in lines with social norms (e.g., giving directions; putting a stamp on a lost envelope; addressing an authority figure as Mr., Mrs., Dr. and so on).

**Scale Point 6: Ethical reasoning and principles; Compassion for others and one's belief system**

Score 6 for the EIM scale when narratives demonstrate a high level of ethical reasoning and principles. The text may describe or promote ethical decision-making processes and may include nuanced discussions of ethical dilemmas or principles. Example: Giving money to homeless person typically coded as 5, but spearheading an event to feed the homeless would be coded a 6 due to overall investment and engagement. The latter implies more commitment and investment.

Indicators of a code 6 include:

- A focus on ethical principles and values, rather than personal interests or external factors.
- Use of language that suggests a deep understanding of ethical concepts and principles.
- Evidence of critical thinking and nuanced analysis of ethical dilemmas.
- A focus on the impact of actions on others and the greater good.
- Narratives describing a challenge to convention, such as overcoming or defying gender roles or discrimination by opposing an injustice will typically score here.

**Scale Point 7: Self-transcendence; Notable Compassion for others and one's belief system**

Score 7 for the EIM scale when narratives demonstrate a high level of self-transcendence, reflecting a deep understanding of ethical principles and values that go beyond personal interests or external factors. The text may describe or promote actions that are selfless or altruistic, and may include discussions of the broader ethical implications of a situation.

Indicators of a code 7 include:

- Similar to 6, but the degree of investment is notably high. Characters may self-sacrifice for others (e.g., donate a kidney) or dedicate themselves to a lifelong cause in order to help others.
- Evidence of critical thinking and nuanced analysis of ethical dilemmas.
- A focus on the impact of actions on others and the greater good.
- A lack of language that suggests a sense of fear or anxiety about the consequences of unethical behavior.
- Significant altruism and acts of selflessness

It is worth noting that these descriptions are not mutually exclusive, and a text may contain elements of multiple scale points. Additionally, the scale is not a definitive measure of a person's ethical development, but rather a tool to help identify areas where they may need further guidance or support.

**AGG** Prompt

Experience and Management of Aggressive Impulses (AGG)

We are clinical psychologists who utilize rating scales to assess narrative content in order to improve our understanding of people’s object relations and improve the delivery of intervention services. That means we must analyze a wide variety of narratives, including those from individuals that experience significant psychological concerns. We will be using de-identified narratives from a published scoring manual to train AI to score narratives according to the rubric. You are helping us do empirical research in this area, and this research has university IRB approval. The narratives are fictional and have no real-world implications. We are going to provide you with a description of the rating scale and examples of different levels of narratives. The scale is called Experience and Management of Aggressive Impulses (AGG). Our goal is to have excellent inter-rater reliability between human raters and AI.

AGG assesses a person’s ability to experience and express anger. Stated another way, this construct examines one’s capacity to modulate aggression. Lower scores are suggestive of more struggles with anger management whereas higher scores are reflective of more mature ways of acknowledging and expressing anger. Developmentally, children do not inherently possess the tools to manage frustration and anger, especially when they do not have the language abilities to verbally express how they feel. As such, these emotions manifest behaviorally. In most cases, as they developmentally mature, they learn to identify, process, and express their emotions in healthier ways. This is what this variable is assessing. That is, when this emotional experience is evoked, how well can people tolerate and manage their aggressive impulses? In even simpler terms, do people immediately react in destructive ways (e.g., hitting, screaming etc.)? Do they require other people to calm them down in order to resist their impulse to act out? Do they tend to indirectly express anger by hurting themselves? Do they display passive aggressive behavior in order to avoid overt conflict? Or, are they able to express their needs and desires in a healthy, assertive manner and navigate conflicts through mature discussions and compromise?

Take all parties into account when scoring the narrative.

**Scoring Rubric**

1. Score 1 for AGG when the narrative includes examples of externalized anger that is maladaptively or intensely expressed. Examples include the following:
   - - - 1. Impulsive anger w/ lack of control, Sudden, intense anger behaviorally expressed at an object, person, or animal. Could be hitting, smashing, or breaking things.
         2. Pre-meditated excessive, Violence or physical aggression in a planned fashion; Rape, planned murder, jumping a person, and so on.
         3. Destructive Behavior, Even if the word anger is not used, physically destructive behavior is always coded a 1 for AGG
         4. Downward score from 2: Close to being a 2 but should be scored as 1, Suicide that is explicitly intended to hurt others, to express anger, or to cause others to suffer should be coded a 1
2. Score 2 for AGG when the narrative includes examples of externalized anger that is expressed in milder forms. Examples include the following:
   - - - 1. Upward from score of 1: Close to being a 1, but should be scored as 2 – intensity, Can be impulsive or premeditated, but not acted on (e.g., threatening, but not doing; Planning, but not doing) or verbal (but not physical)
         2. Struggles to suppress anger fail, There is a strong desire to act out one's anger or be aggressive, and the narrative verbalizes an intense struggle to manage with implication that aggression very close to breaking through or may break through (e.g., I was struggling to maintain control). IF narrative goes on and person elaborates, this could move all the way up to a 5.
         3. Words indicating a loss of control around anger, Words or phrases that imply an intensity of anger that is excessively difficult to control (e.g., Rage; Enraged; Bursting with anger; I exploded with anger) can imply a 2 if not mitigated.
         4. Intense Verbal Conflicts, Intense verbal confrontation (e.g., yelling; screaming) during a disagreement is often coded as a 2 (if it becomes physical, then should code 1).
         5. Downward score from a 3: Close to being a 3, but should be scored as 2, Particularly intense suicides that are gruesome and particularly self-aggressive (e.g., light self on fire) and intend to cause the heightened pain experienced by the self can be coded a 2 (note suicide with intent to hurt others should be coded a 1).
3. Score 3 for AGG when narratives include descriptions that involve passive-aggressive acts, less severe (i.e., not abusive) forms of anger, and anger without action
   - - - 1. Upward 2 - Milder rule breaking, Conflict and bad behavior that is less directly aggressive; an intense argument is a 2, but spreading rumors, gossiping, complaining about someone and so on is a 3.
         2. Anger thoughts, feelings, or sensations, Expressions of anger involving feeling angry/upset, physical sensations (heart racing; turning red), or thoughts (imagined acts or fantasizing aggression); verbalized anger words, such as frustration, anger, irritation, and so on code here if unmitigated (i.e., no turning point). Individuals experience and describe anger, but do not direct anger at others or objects through physical actions.
         3. Passive Aggressive Acts/ Statements, though passive aggressive acts are done and directed toward someone, they are coded a 3, because they are indirect.
         4. Self-Harm, Attempted suicides are coded a 3; non-suicidal self-injury is coded a 3
         5. Failure to protect the self from harm is a 3 (but if the harm is sufficiently severe to be described as abusive code as 2).
         6. Spoilage or Reversal, A story in which anger is managed well, that suddenly reverses at the end to suggest that prior management was fake or limited in effectiveness, can be scored a 3 if degree of spoilage exceeds what would allow for a 4.
4. Score 4 for AGG when the narrative contains no anger content. Also, score a 4 for AGG if the narrative is an between a score of 3 and a score of 5.
   - - 1. Limited in use; typically given when one person in a narrative shows a mixture that is roughly equal between positive and negative expressions and the narrative doesn't resolve in a manner that allows us to go one way or the other.
5. Score 5 for AGG when the narrative include descriptions of hostile, angry or aggressive impulses that are internalized/warded off/defended in a non-physically or self-harming way. Examples include:
   - - - 1. Behavioral Control, The person feels anger, but controls it (Even if internalizes or swallows their pride or feelings).
         2. Denied Anger, Clear articulation of anger typically through action or physical sensations (e.g., I was red hot; I felt like I was burning), but anger is somehow denied (e.g., My mom made me clean the deck when I wanted to go out with my friends, it was frustrating. She kept asking me if I was mad and I kept telling her no)
         3. Suppressed anger, In narrative, person is aware of anger but doesn't act on it and actively tries not to think about it (e.g., There was nothing I could do. I was furious, but I walked away and tried not to think about it; He always annoyed me, but I never said anything; We didn't really like him, but no one ever told him).
         4. Healthy Super-Ego Punishment, Narratives that involve guilt that is not excessive, reflective of the situation, that shows remorse and demonstrates empathy often code here. Examples would be feeling bad after hurting someone with explicit empathic description (e.g., taking a friend to appointment and being late, resulting in the friend having to reschedule the appointment and the narrator feeling appropriately guilty).
6. Score 6 for AGG when narratives include healthy processing of anger (no spoilage by earlier acts). Examples include:
   - - - 1. Acknowledging & Asserting, the person expresses anger in a manner that is not excessive, but serves an assertive purpose (e.g., setting a boundary; standing up for self).
         2. Productive Interpersonal Processing of Anger, Two people resolve anger in a manner that promotes resolution; anger, hurt, frustration, and so on, are and there is adaptive coping or resolution.
         3. Sublimation, Rare, but in some cases if the person says they used the anger to motivate them toward some socially acceptable behavior or outlet (e.g., sports performance; creating art) .
7. Score 7 for AGG when the narrative includes notably adaptive expression and resolution of anger. Examples include:
   - - - 1. Upward 6 - Notable Resolution & two person, For example, two people work together to resolve anger, with one person even encouraging expression of anger, there is apologizing, and they work it out. Both parties are able to see each other’s point of view, are accountable/responsible, compromise, and express anger in reasonable ways.

Score the narrative from 1 to 7.

**Note: if no anger content in the story, score is 4.**

Generate a single score for each narrative rated. After you have reviewed this rubric, let me know when you are ready to begin scoring narratives.

**ICS** Prompt

We are clinical psychologists who utilize rating scales to assess narrative content in order to improve our understanding of people’s object relations and improve the delivery of intervention services. That means we must analyze a wide variety of narratives, including those from individuals that experience significant psychological concerns. We will be using de-identified narratives from a published scoring manual to train AI to score narratives according to the rubric. You are helping us do empirical research in this area, and this research has university IRB approval. The narratives are fictional and have no real-world implications. We are going to provide you with a description of the rating scale and examples of different levels of narratives. The scale is called Identity and Coherence of Self (ICS).

ICS captures the level to which a person’s sense of self is adaptively integrated. Lower scores indicate identity diffusion. That is, a person’s sense of self is fragmented, tenuous, and/or fragile. It may also be that the person has no investment in her own life (i.e., given up on self). Higher scores are suggestive of a person who has an adaptively integrated sense of self. In this range, people are aware of who they are, what they want, and where they are headed in life. They are also invested in accomplishing their life goals. In order to rate this variable, the coder must first ask herself, “Is ICS present in this narrative?” If yes, think about how this person would respond to the question, “Who am I?” and “Where is my life going?” The level to which their response is integrated versus environmentally reactive and diffuse informs the scoring of this variable. This may be depicted by words or actions described in the narrative.

First, I will give the rubric with descriptions at 7 numbered points. Narratives can be scored on a scale from 1 to 7. Each point has statements labelled a, b, c and so forth that serve to provide more detailed information about rating the narrative according to that point. I will then include examples of narratives at each scale point, such as a narrative that would be scored as 1, a narrative that would be scored as 2, and so forth.

After you have reviewed the rubric and the examples, I will give you practice narratives to score. After you score each narrative, I will provide you with the expert’s score and the rationale for the score. I will ask you to update your scoring criteria to improve accuracy for future scoring.

After completing the practice scoring, I will ask you to write a very detailed prompt to enable AI to accurately score narratives with little-to-no additional training. The prompt should contain explicit instructions that guide AI to score the narratives accurately for ICS. You will then create detailed language for each scale point and provide whatever additional instructions should be considered for accurate scoring.

ICS Rubric

Is ICS present? If no, then score 5; if yes, then use the following scale below to assign a score to the narrative.

1. fragmented self, dissociative, multiple personalities
   1. I've completely lost myself; I have no idea who I am
   2. Complete retrograde amnesia; not knowing if you are you or another person
   3. Turning from one person into another person in a literal way
   4. Suicidal or self-harm in dissociative or fragmented states
2. Upward extension of a 1 (e.g., lost) or instability in the face of challenge that leads to more pathological loss of sense of self or pathological acting out behavior (downward extension of 3)
   1. Brief dissociative experiences (e.g., not in my right mind; lost control; lost myself; lost mind); Feeling "as if" you are someone else (but knowing you are not; it's like someone else did it)
   2. Severe loss of control reaction (e.g., flying into a rage; an unfamiliar calmness came over me; I did it and I didn't even know I was doing it; It was like being someone else).
   3. Notable "loss of self" reactions in response to status changes (e.g., I have no idea who I am without her; He couldn't even begin to fathom a life in which he wasn't a banker).
   4. Suicide or self-harm in the form of "giving up"
3. Fragile or reactive self (not as bad as 2), emptiness lack of self. Different from 2 in that is less severe and less acted out; more of a cognitive experience (e.g., self-criticism; considering self-harm)
   1. Inconsistent self-statements, but they are not dissociative (e.g., I go back and forth; I'm unstable)
   2. Statements that suggest milder levels of "giving up," such as overreactions at inability to overcome a situation or challenges (e.g., end of my rope; can't take it anymore; overwhelmed) or loss of sense of self-efficacy in response to set backs (e.g., I am a failure).
   3. Milder statements of "loss of sense of self" in reaction to status changes (e.g., After my husband left, I felt empty; If I'm not a lawyer, then who am I?)
   4. Milder statements of "loss of sense of self" reactions frustrations that indicate loss of confidence in one's judgments (e.g., afterwards, I thought I don't know who these people are at all; When I failed, I wondered why I ever thought I would succeed?).
   5. Milder statements of "loss of sense of self" reactions that result in a shift in affect/self-view (e.g., It didn't go as expected, and I just felt so ashamed; They let me down and I felt empty; After the feedback, I felt small and powerless).
   6. Statements that reflect a "shaken" or "off kilter" identity; (e.g., I was new, and I felt different from everyone else [with no resolution]; I felt out of place; I didn't feel like I knew who I was after I moved).
   7. Triggered and responding with toward shame, sudden loss of goodness of self, or contemplating self-harm (triggered and actually engaging in self-harm is coded a 2).
   8. Clear foreclosure; meaning evidence of simply accepting the goals, interests, purposes, of others as if they were one's own (e.g., accepting another's goals over one’s own; becoming a doctor to please parents).
   9. Clear diffusion; the person is so open to the immediate situation that they lack any clear goals or sense of organizing direction.
   10. Notable negative shift in sense of self in relation to major event (e.g., break up, loss of job, end of school)
   11. Unrealistic self-questioning (e.g., self-denigration, perfectionistic thinking, perception of inadequacy) in the context of challenges to one's efficacy, unexpected, changes in status, or frustration in pursuit of goals/experience of setbacks.
4. Downward extension of 5, at least some goal/purpose is described, but described in a vague or unelaborated fashion.
   1. Identity related theme, but unclear if adaptive or maladaptive (e.g., trying to contemplate the future; ambivalence about goals) with no sense of adaptive resolution.
   2. Undoing; purpose mentioned followed immediately by block (e.g., He wants to write, but he's not sure he can; he wants to go, but he feels trapped).
   3. Vague references to change without efficacy statements or overly passive expressions (e.g., He wants things to be different; they hoped things would change; she doesn't want it to be this way; he didn't like where his life was at; she didn't like where things took her).
   4. Theme of purpose is raised, but agency seems low, or the person is passive in their approach (e.g., he wished for a change, but didn't think there was anything he could do; she wanted to achieve greatness, but it was all out of her control).
   5. "loss of self" reaction with a clear implication of intent to deal with it (e.g., I am trying to figure out who I am after the accident; she had to figure out who she was without him). The person has an "active" voice indicating agency, even if no resolution as of yet.
   6. Identity related theme, poorly developed (e.g., he wants to do good some day; he thinks things will change)
5. Default code OR group context identity.
   1. Identification with a group goal without elaborating on how goal is unique within the individual (e.g., Like everyone else, I wanted to go to college)
   2. Communal identity
   3. Greater emphasis on group purposes than one's own purpose or role within that group.
6. Sense of realistic purpose with elaboration of needs, wants, goals, future and/or focus on navigating challenges associated with identity.
   1. Describes self/character as driven, ambitious, or other words implying achievement orientation and/or goal-directed planning.
   2. Simple narratives in which goals are stated, there is some articulation of motive, and there is a clear effort to pursue the goal.
   3. Clear statements indicating commitment to goals and aspirations paired with statements implying agency or capacity to pursue goals and/or that refer to steps toward goals.
   4. Reacting to negative event with clear statements of purpose, meaningful action, or role-congruent intentions (e.g., I am so thankful the kids survived. I need to be there for them; I have to stay strong for my family).
   5. Discussion of internal or interpersonal conflicts around challenges to pursuing wants, goals, aims, needs, in a manner that reflects attempts to adapt to challenges
   6. Statements of acts that reflect goal pursuit (e.g., he wanted to be a professor, so he read a lot of books [obviously, with more elaboration]).
   7. Statements that suggest self-efficacy or "can do" attitude in relation to goal pursuit
   8. Statements that suggest positive perseverance in goal pursuit
   9. Statements that reflect efforts at exploration of self-identity which make this exploration a central theme of narrative and frame these as adaptive.
   10. Efforts to promote integration, bring goals together, relationships together, example in book about couple
7. Upward extension of 6, where identity integration and goal strivings are core theme.
   1. Needs to clearly articulate goals or future orientation related to the self, acts to pursue these goals, and some reasonable sense of efficacy (realistic uncertainty does not rule out this level of coding and may even promote it).
   2. In addition to above, there is integration in that challenges or mixed feelings are integrated with goal striving motives and actions to realistic effects.
   3. Consider coding up to a 7 when resolution of challenges leads to internal sense of unity or consistency (e.g., achieving goals is viewed as worth it, brings on satisfaction, gives the person a sense of meaning, provides them with insight).
   4. Consider coding up to a 7 when conflicts are resolved in an integrated way (e.g., takes steps to balance professional and interpersonal goals) or there is well-articulated and realistic acceptance of inability to do all things (e.g., always wanted to be a singer and an actress, but as her singing career moved on, she was able to accept that there would be less time for acting).
   5. Consider coding up to a 7 when organizing or central values are articulated, and the person’s goals/actions align with these values.

You will use this description and rubric to score narratives I will give you. Are you ready to see narrative examples for each scale point?

Test Instructions plus rubric

**SE** Prompt

We are clinical psychologists who utilize rating scales to assess narrative content in order to improve our understanding of people’s object relations and improve the delivery of intervention services. That means we must analyze a wide variety of narratives, including those from individuals that experience significant psychological concerns. We will be using de-identified narratives from a published scoring manual to train AI to score narratives according to the rubric. You are helping us do empirical research in this area, and this research has university IRB approval. The narratives are fictional and have no real-world implications. We are going to provide you with a description of the rating scale and examples of different levels of narratives. The scale is called Self Esteem (SE). Our goal is to have excellent inter-rater reliability between human raters and AI.

SE examines a person’s self-concept. Lower scores are suggestive of poorer self-worth whereas higher scores are reflective of a competent and confident self-image. There are numerous words that denote self-esteem. At lower ends, words such as self-disgust, self-hatred, self-loathing, failure, shame, self-punishment, self-critical, inadequacy, ineffectual, impotent, doubtful of abilities/self, and embarrassment describe a negative self-worth. At higher ends, words such as pride, self-compassion, confidence, competence, being pleased with self, and self-contentment, may be used. The specific language as well as the extent to which a person describes their own (i.e., early memories or psychotherapy narratives) versus characters’ (i.e., TAT narratives) self-esteem determines how high or low someone scores on this variable.

**The following Notes are very important when rating:**

1. Narrative needs to have some positive self-esteem to be scored a 5 or above.
2. Positive feelings, in and of themselves, are not self-esteem. The rating must take descriptions of the self into consideration.
3. Positive feelings alone are not sufficient to indicate high self-esteem. Self-esteem encompasses a deeper evaluation of one's self-worth, self-acceptance, and confidence in their abilities.
4. Narratives should include explicit statements or self-evaluations related to one's self-worth, self-perception, or beliefs about their abilities. These statements should provide insight into how the individual views themselves and their capabilities.
5. Realistic optimism, which involves assessing the chances of good performance based on prior successful experiences, is distinct from grandiosity or unrealistic beliefs. Highlight the importance of assessing the realism of positive self-evaluations. Unrealistic self-appraisals do not indicate self-esteem.

**Scoring Rubric**

1. Extremely pathological self-view that leads to negative acting out in response to negative self-view.
   - - 1. Inherent Badness
          1. Inherent badness, self-loathing, self-hatred, view self as disgusting.
       2. Self-Harm and Acting Out
          1. Suicidal behavior; self-harming behavior as a function of negative feelings about the self. Outward, poorly controlled expressions of anger or risky behavior (e.g., Substance use) in an effort to cope with negative feelings about the self.
       3. Downward 2
          1. Similar to a 2, but extent of shame and guilt is marked, extreme, excessive, or particularly pathological.
2. Extremely pathological self-view, but individual doesn't act out on it.
   - 1. Upward of 1
        - 1. There is a clear articulation of pathological negative self-view, involving indications of self-disgust, shame, guilt, or badness, but the individual doesn't act this out toward self or toward others; it's just a negative self-attitude. If the narrative rises to the level of self-hatred, consider rating it a 1.
3. Negative self-view, but less intense, covering various forms of experiencing one's self as inadequate, not measuring up, unworthy, or inferior.
   - 1. Upward 2 - less pathological Less intense negative self-view (e.g., inadequate as opposed to self-disgust)
     2. Inferiority, feeling inferior or not measuring up relative to self-expectations or in one's evaluation of what others are capable of; an actual vs. ideal self-discrepancy or an actual vs. should self-discrepancy. Keywords involve: inferior, awkward, insecurity, out of place, not fitting in, doubtful; hopeless; feeling defeated
     3. Grandiosity, Clearly unrealistic grandiosity is a 3; this includes saying that people can do something without any prior experience or practice (e.g., though he's never played the violin before, he picks it up and his fingers just know where to do) or unrealistically excessive evaluations of performance (e.g., I was the best ever [vs. I was the best player on the team]). Grandiosity is different from realistic optimism. Realistic optimism is about assessing chances of good performance because of prior experiences that show success is possible.
     4. Self-doubt or Self-Conscious, Expressions of self-doubt or self-consciousness through explicit statements or self-evaluations (e.g., I can't do this; this is too hard; I'm not good at this; I'll fail)
     5. Perfectionistic Strivings, Clear self-ideal self discrepancies, expressions that indicate a desire for perfection or discontent that one is not perfect. Statements indicating that one's efforts are never enough.
     6. Downward of a 5 – spoilage, The narrative may contain positive self-statements, but the positive self-statements set up the theme of a big fall or spoil (e.g., I was proud of my bike and excited to show it to the other kids, but they all laugh, ridiculed me, and I was left feeling small and powerless).
4. Not present; there is no evidence of or reference to self-esteem in the narrative
5. Healthy expression of self-esteem, there may be negative feelings but there are also some positive evaluations or realistic evaluations. There must be an expression about the self, not simply positive feelings. Feeling good is not enough if it does not directly relate to self esteem
   - - 1. Positive self-statements, There must be some clear positive self-statement, this may involve pride, expressions of confidence or perseverance (e.g., I kept going; I believed I could do it), and these are not spoiled by negative statements.
       2. Positive Self Evaluation/ description, Statements like "I was a good worker;" "I was good at math"; "I was good at the piano" and so on.
       3. "Can do" attitude or implicit self-confidence, Statements that imply hope, confidence one can do something; giving self a pep talk; taking on a new challenge; needs to be some resolution where the person feels positively in relation to completing the task or growing or moving forward in some way.
       4. Mixture or realism, Some positive statements are also present, but the narrative includes a few statements involving negative self-evaluation or lack of confidence. Negative self-statements are built into a narrative of growth or overcoming challenges. Positive feelings occur after overcoming challenges or resolving conflicts between negative and positive.
6. Solely positive in a realistic manner
   - 1. Upward 5 - No negative, Similar to a 5, but there is less of a focus on the negative and a clear focus on the positive.
     2. Achievement Theme, The primary focus on the narrative is that the individual achieves something and feels good about it. Though there may not be a lot of positive self-statements, the theme of the entire narrative involves overcoming or achieving and positive self-feelings as a result.
7. Solely positive and primary theme Upward 6 - primary theme
   - 1. Same as a six code, except that it is as if the main theme of the narrative was self-esteem.
     2. Negative reactions without negative self, the theme is about achievement or pride, but the story contains negative events that involve a short-term negative reaction that don’t hurt self-worth and the character responds to adaptively (e.g., I would say, I will get this next time; I took a break and came back and kept going). The story tends to close with sense of achievement, pride, sense of accomplishment, positive evaluations from others, and so on.

Please review the rubric and I will give you narratives to score.
